# Reducing noise in radioactive iodine activity selection: the utility of an online clinical calculator

**DOI:** 10.1530/EC-24-0299

**Published:** 2024-10-09

**Authors:** Ayanthi Wijewardene, Matti Gild, Lyndal Tacon, Venessa Tsang, Anthony J Gill, Anthony Robert Glover, Mark Sywak, Stan Sidhu, Bruce Robinson, Paul Roach, Geoffrey Schembri, Jeremy Hoang, Roderick Clifton-Bligh

**Affiliations:** 1 Department of Endocrinology, Royal North Shore Hospital, Sydney, Australia; 2 Faculty of Medicine, The University of Sydney, Sydney, Australia; 3 Department of Anatomical Pathology, NSW Health Pathology, Royal North Shore Hospital, Sydney, Australia; 4 Cancer Diagnosis and Pathology Group, Kolling Institute of Medical Research, Royal North Shore Hospital, Sydney Australia; 5 Department of Endocrine Surgery, Royal North Shore Hospital, Sydney, Australia; 6 Department of Nuclear Medicine, Royal North Shore Hospital, Sydney, Australia

**Keywords:** radioactive iodine, risk stratification, differentiated thyroid cancer, recurrence

## Abstract

**Background:**

Noise, an unwanted variability in judgment, is ubiquitous in medicine, including in the prescription of radioactive iodine (RAI). Building upon our recently developed predictive risk model, we created an online clinical support tool to facilitate the translation of our model into clinical practice. The aim of this study is to assess the utility of an online clinical support tool to reduce noise in the treatment for patients with differentiated thyroid cancer (DTC).

**Methods:**

The tool was accessible via weblink or a QR code. Activity recommendations were applied to the calculator’s four risk categories: 0 GBq for very low risk, 1 GBq for low risk, 4 GBq for intermediate risk, and 6 GBq for high risk. The tool was applied prospectively to 103 patients who received RAI at Royal North Shore Hospital between 2021 and 2022 and retrospectively to 393 patients treated with RAI between 2017 and 2021.

**Results:**

A significant difference was observed in administered activity between the 2021–2022 and 2017–2021 cohorts in patients stratified as intermediate risk (median activity 3.95 GBq, interquartile range 2.03–4.04 vs 4 GBq, 4–4) and high risk (4.07 GBq, 3.95–5.7 vs 6 GBq, 6–6) with *P-*values of 0.01 and <0.01, respectively. No difference was seen in low-risk patients (2.01 GBq, 1.03–3.98 vs 1 GBq, 1–4, *P* = 0.30). Additionally, no clinically significant recurrence was observed between the two cohorts (6.6% vs 4.5%; *P* = 0.628).

**Conclusion:**

Optimal risk classification and activity recommendation continue to be established. Our data suggest that providing risk stratification and activity recommendation in an easy-to-access online tool can reduce noise and variability in activity prescription for patients with DTC.

## Introduction

Radioactive iodine (RAI) is frequently administered in the management of differentiated thyroid cancer (DTC) to reduce the risk of recurrence or treat persistent disease following a total thyroidectomy. The recommendations for RAI activity are guided by international guidelines, including the 2015 American Thyroid Association (ATA) Management Guidelines for Adult Patients with Thyroid Nodules and Differentiated Thyroid Cancer (1). However, implementing new guidelines into clinical practice can face significant barriers, resulting in inconsistent practice. Noise, an unwanted variability in judgment (2), is a common occurrence in medicine, and can result in unpredictable treatments, and associated increased costs. In medicine, noise is ubiquitous. Variability in thyroid cancer management is even observed in a multidisciplinary team setting, with discordant recommendations not explained by clinical variables (3). Delays in thyroid cancer surgery can result in significant morbidity and mortality (4), and in addition cause significant psychological burden to patients (5).

In thyroid cancer, the selection of RAI activity administered can have significant implications for patient outcomes and costs. First, patients who receive higher RAI activities have longer length of stay in hospital, requiring admission of two nights for treatments ≥4 GBq compared to patients who receive 1 GBq who are only admitted for one day. Secondly, higher activities of RAI therapy are associated with increased risk of complications (6). Finally, patients treated with inappropriately low activities of RAI therapy in the presence of high-risk features are at risk of needing a second treatment, which can cause undue stress for patients and are associated with healthcare costs (7).

Building upon our recently developed predictive risk model (8), we created an online clinical support tool to facilitate the translation of our model into clinical practice and overcome the implementation barriers associated with new guidelines. We expanded our predictive risk model to include recommendations for RAI activity. Current guidelines offer only broad activity recommendations, which can be challenging for individual clinicians to interpret (1). To our knowledge, this is the first online calculator to risk stratify patients with DTC.

The aim of this study is to assess the utility of an online clinical support tool to reduce noise in the treatment of patients with DTC (8).

## Methods

### Extension of predictive risk model to include activity recommendation

We developed a predictive risk model that extended the ATA guidelines by including histological extrathyroidal extension (ETE) and stimulated thyroglobulin (sTg). The model was validated in two retrospective cohorts (8). Utilizing current literature and clinical expertise, we expanded our predictive risk model to recommend RAI activity, employing a four-tiered risk classification: 0 GBq for very low risk; 1 GBq for low risk; 4 GBq for intermediate risk; and 6 GBq for high risk (Table 1).

**Table 1:** Algorithm for RAI activity used in online clinical support tool to guide activity selection in differentiated thyroid cancer.

Very low risk (0 GBq)	Low risk (1 GBq)	Intermediate risk (4 GBq)	High risk (6 GBq)
P1a (<2 cm and confined to thyroid)	BRAF mutated intrathyroidal tumor ≤4 cm	Aggressive risk histology (Hürthle cell, tall cell, columnar, and DSV) and tumor size ≥4 cm.	Distant metastasis
AND	OR	OR	OR
sTg <1 µg/L.	Tumor size ≥4 cm	LN ≥5 lymph node involvement or ≥3 cm greatest diameter.	FTC extensive vascular invasion (≥4 foci)
AND	OR	OR	OR
No lymph node involvement	Multifocal micro PTC (TTD < 1 cm)	Vascular invasion	TERTp mutation
AND	OR	OR	OR
No ETE	Minimal invasive encapsulated FTC with <4 foci of vascular invasion	FTC/Hürthle cell 4 cm	Widspread ETE into soft tissue and muscle
AND	OR	OR	OR
No vascular invasion	sTg <10 µg/L	R1 (microscopic involvement of margins).	Extranodal extension and ≥ 3 LN
AND	OR	OR	OR
Absence of lateral LN compartments on neck US	LN (N1a) and <5 disease	N1b and <5 LN	R2 (incomplete tumor resection)
AND	OR	OR	OR
Complete tumor resection	Aggressive risk histology ( Hürthle cell, tall cell, columnar, and DSV) (if none of the above features and <4 cm)	ETE out of perithyroidal fibroadipose tissue	sTg ≥ 30 µg/L
		OR	
		sTg ≥10 µg/L	

DSV, diffuse sclerosing variant; ETE, extrathyroidal extension; FTC, follicular thyroid carcinoma; LN, lymph node; PTC, diffuse sclerosing variant ; R, residual tumor; sTg, stimulated Tg; TTD, total tumor diameter.

### Development of an online support tool

The online tool was developed on the NSLHD REDCap database by AW.

It consisted of clinical and histological variables linked by branching logic to assign a risk outcome: very low, low, intermediate, and high risk. An activity recommendation was provided for each risk category. For practicality, since sTg is not readily available prior to activity selection, a 6-week postoperative unstimulated Tg was utilized for the online calculator.

In the prospective cohort, if an unstimulated Tg was available, a value of <1 µg/L was required for stratification as either very low or low risk, <10 µg/L for intermediate risk, and ≥10 µg/L for high risk. In the retrospective cohort, an unstimulated Tg was not available, and an sTg was utilized as outlined in Table 1.

TERT was not included in the online clinical support tool due to its limited testing availability.

The tool was accessible via weblink or a QR code: https://rnsapmredcap.nslhd.health.nsw.gov.au/redcap/surveys/?s=PCYF944RARRTRFFM.

### Clinical trial

All patients aged ≥18 years with DTC referred for RAI therapy following a total or completion thyroidectomy from August 2021 to July 2022 at Royal North Shore Hospital (RNSH), Sydney, Australia, were prospectively assessed using the online clinical support tool. As part of the tool, clinicians were surveyed to assess their agreement with activity recommendations. The tool was applied retrospectively to patients already treated with RAI therapy from August 2017 to July 2021. Follow-up was at the discretion of the treating clinician, and the usual clinical follow-up involved 6- or 12-month reviews with biochemical and radiological testing. Biochemical testing included both serum unstimulated Tg and Tg antibody (Tg Ab), and structural assessment with neck ultrasound and whole body iodine scans (WBS) and/or fluorodeoxyglucose PET (FDG PET) if indicated. Follow-up data were obtained from a prospectively maintained RNSH Thyroid Cancer Database and electronic medical records. Recurrence was defined as either biochemical or structural disease in patients who had no evidence of disease on post-treatment WBS. Persistent disease was defined as patients who had evidence of uptake in the neck (excluding thyroid bed) or distant metastasis at the initial post-treatment WBS. The Human Research Ethics Committee at Northern Sydney Local Health District approved the study (ETH01168/STE02305).

### Statistical analysis

Descriptive data were expressed as mean (± s.d.) for normally distributed data and as frequencies and proportions for categorical variables. Mann–Whitney tests were used to compare medians between two groups. All median values were expressed with interquartile range (IQR). The chi-square test was used to compare categorical groups and differences in recurrence outcomes. An *F*-test was used to compare the variability in activity prescription between the two-time points. A *P* value of ≤0.05 was considered statistically significant. Statistical analyses were conducted using Statistical Package for Social Sciences (SPSS) version 26 (SPSS Inc.). Figures were created using GraphPad Prism version 8.0.0 for Windows (GraphPad Software).

## Results

### Patient characteristics

The tool was applied prospectively to 103 patients who received RAI therapy at RNSH. Complete follow-up data were available for 89 patients. The mean age of the prospective cohort was 51.2 years, with 60 out of 89 patients (67.4%) being female. As a comparator, the tool was retrospectively applied to 393 patients treated with RAI between 2017 and 2021. The mean age of the retrospective cohort was 50 years, with 281 out of 393 (71.5%) being female. There was no difference in age, gender, histological characteristics, ETE, and lymph node involvement between prospective and retrospective cohorts (Table 2).

**Table 2:** Clinical and histological baseline characteristics of prospective and retrospective cohorts.

	Prospective cohort, *n* = 89	Retrospective cohort, *n* = 393	*P* value (<0.05)
Age (mean)	51	50	0.80
Female (*n*, %)	60 (67)	281 (72)	0.44
Histology (*n*, %)			0.06
Classical papillary thyroid cancer	57 (64)	252 (63)	
Follicular variant PTC	9 (10)	78 (20)	
Tall cell	2 (2)	9 (2)	
Diffuse sclerosing	1 (1)	7 (2)	
Follicular thyroid cancer	11 (12)	23 (6)	
Hürthle cell	9 (10)	24 (6)	
Tumor size (mean)	31 mm	24 mm	0.50
Extrathyroidal extension			0.10
Confined to thyroid	49 (45)	169 (43)	
Minimal	37 (42)	204 (52)	
Gross	3 (3)	20 (5)	
Lymphovascular invasion	58 (64)	191 (49)	<0.01
BRAF^V600E^			0.29
Positive	62 (79)	168 (73)	
Negative	16 (21)	63 (27)	
Not available	11	121	
Lymph node involvement			0.09
≥5	20 (22)	54 (14)	
<5	33 (37)	181 (46)	
No	36 (41)	158 (40)	
Distant metastasis	0 (0)	7 (2)	0.20

### Risk classification by online clinical support tool

In the 89-patient prospective cohort, our online clinical support tool stratified 15 (17.5%) were classified as high risk, 57 (64%) as intermediate risk, 15 (17.5%) as low risk, and 1 (1%) as very low risk. The majority of clinicians, 79 out of 89 (88.8%), agreed with the risk classification and activity recommendation.

During the 2-year period, 31 treating clinicians used the tool, with all treatments administered through RNSH. However, nine different physicians did not adhere to the recommended activity from the online calculator for ten patients. Of these patients, one was stratified as very low risk, four as low risk, three as intermediate risk, and two as high risk.

Out of the ten patients, seven received a higher activity than recommended: five patients received 4 GBq and two received 6 GBq. The remaining three patients received a lower activity than recommended: one declined RAI therapy despite a recommendation for 4 GBq and the other two, who were stratified as high risk, received 4 GBq. One of the high-risk patients had an unstimulated Tg level of 8.87 μg/L prior to treatment and showed persistently elevated Tg levels 12 months post-treatment. The other high-risk patient had FTC with > 4 foci of vascular invasion. The clinical characteristics of the patients who did not receive the online support tools recommended activity are reported in Table 3.

**Table 3:** Clinical characteristics of the 10 patients in the 2021–2022 cohort who did not receive the tools recommended RAI activity.

Age (median, IQR)	57.7 (IQR 43–73)
Female, *n* (%)	7 (70)
Histology, *n* (%)	
PTC	6 (60)
FTC	3 (30)
Hürthle cell	1 (10)
Largest tumor size (median)	29 (IQR 12.5–52.5)
Lymph node (LN), *n* (%)	
No LN	3 (30)
<5 LN	5 (50)
≥5 LN	2 (20)
N1a	4 (57)
N1b	3 (43)
Extrathyroidal extension (ETE), *n* (%)	
No ETE	6 (60)
Minimal	4 (40)
Gross ETE	0 (0)
Vascular invasion, *n* (%)	3 (30)
Tg available, *n* (%)	2 (20)
BRAF^V600E^, *n* (%)	8 (80)
Surgical margins, *n* (%)	
R0	9 (90)
R1	1 (10)
Distant metastasis	0 (0)

In the retrospective cohort of 393 patients, 77 (20%) were classified as high risk, 218 (55%) intermediate risk, 85 (22%) low risk, and 13 (3%) very low risk.

### Variability in RAI activity administration

**Figure 1:**
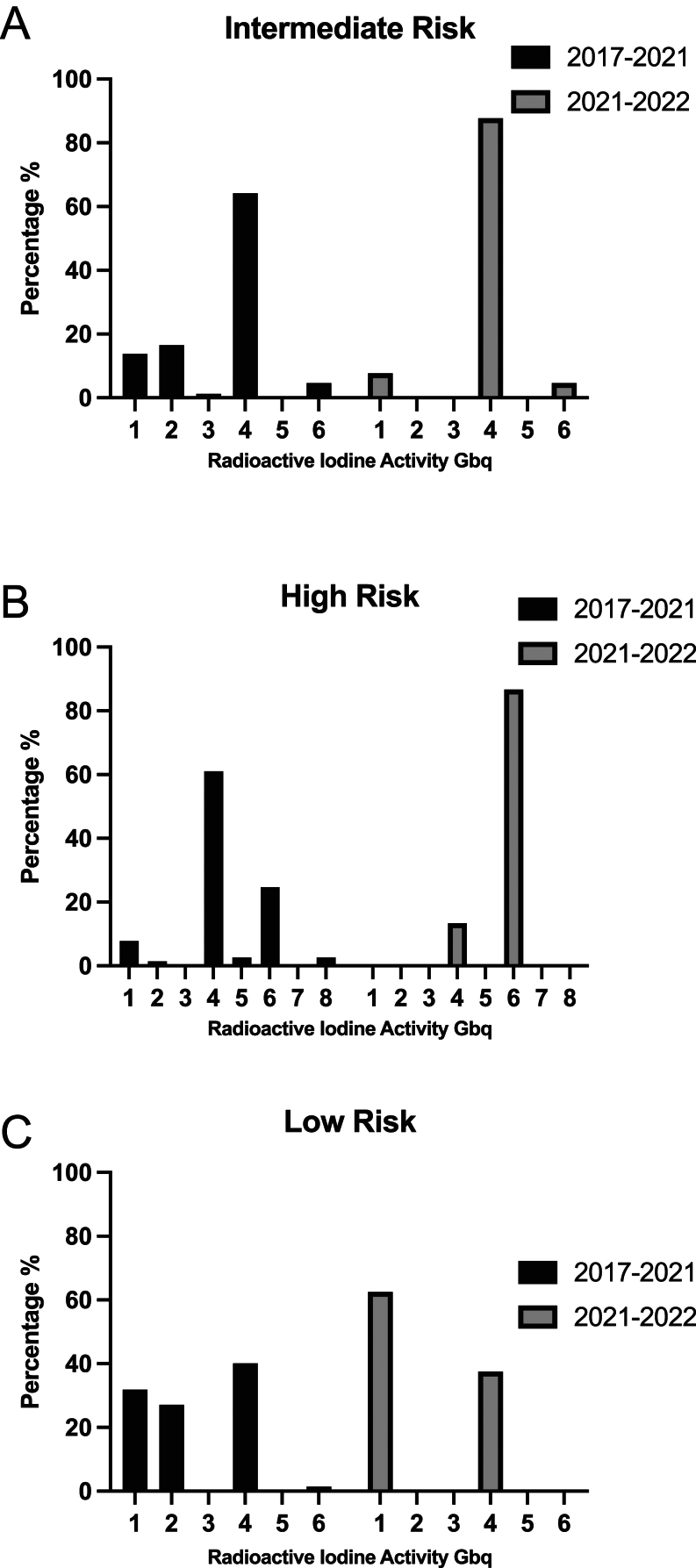
Histogram of RAI activity administration in (A) intermediate-, (B) high-, (C) low-risk categories in both 2017–2021 vs 2021–2022 cohorts.

**Figure 2:**
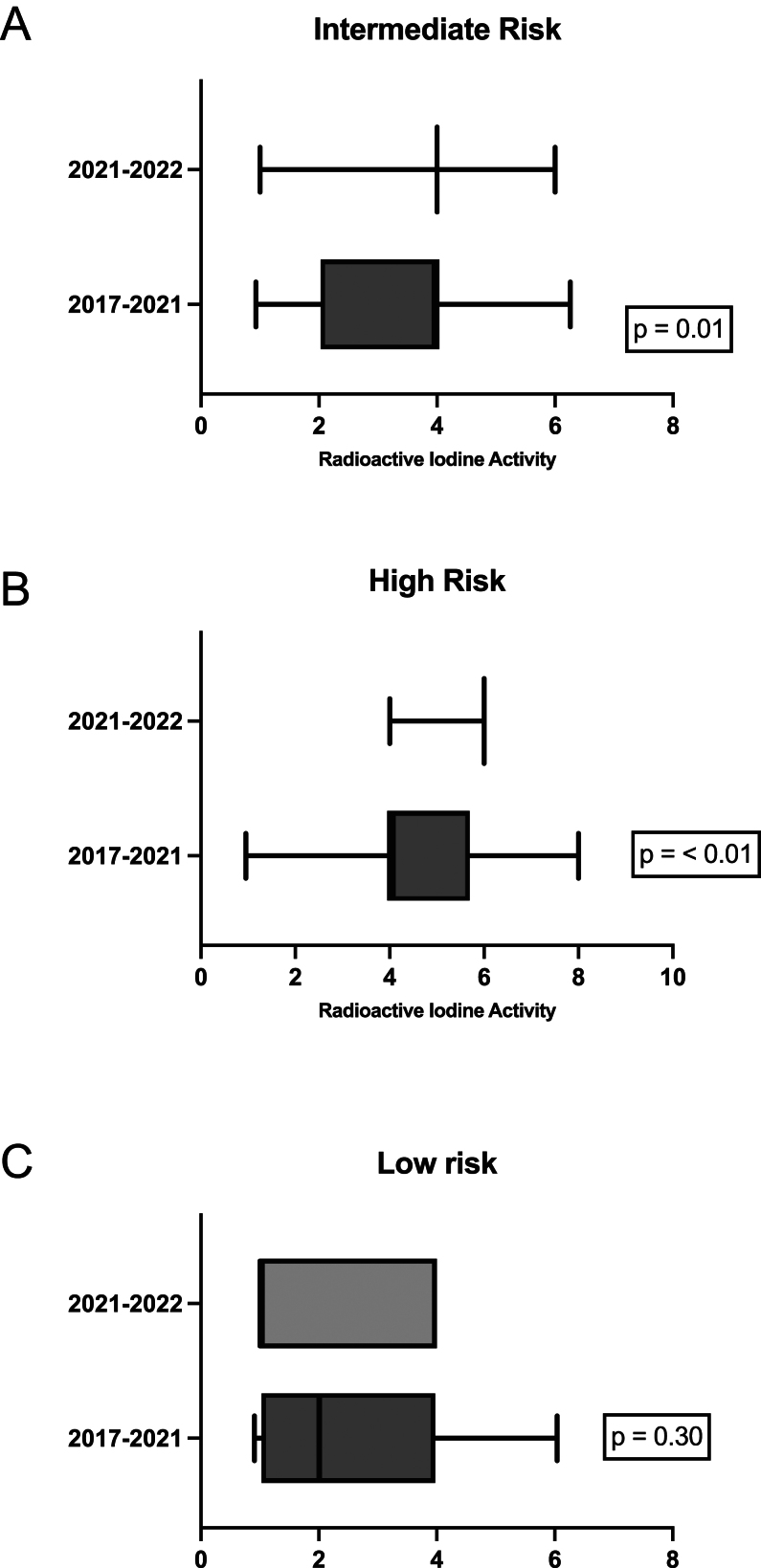
Box-and-whisker graph demonstrating the greater range of activity selection prior to the tool implementation in (A) intermediate-, (B) high-, (C) low-risk categories.

Greater variability was seen in RAI activities administered to patients classified as intermediate risk by our calculator in 2017–2021, with 14% receiving 1 GBq, 17% receiving 2 GBq, 64% receiving 4 GBq, and 5% receiving 6 GBq. In contrast, among those treated in 2021–2022, 8% of intermediate-risk patients received 1 GBq, 88% received 4 GBq, and 5% received 6 GBq (Fig. 1A). The median activity for those stratified by the calculator as intermediate risk was 3.95 GBq (IQR 2.03–4.04) in the 2017–2021 cohort vs 4 GBq (IQR 4–4) in the 2021–2022 cohort. A significant difference in variance was observed in administered activity between the two cohorts (Fig. 2A, *P* = 0.01).

Similar variability was seen in RAI activities administered to patients stratified as high risk by our calculator from 2017 to 2021, of whom 8% received 1 GBq, 1% received 2 GBq, 61% received 4 GBq, 24% received 6 GBq, and 2% received 8 GBq, with a median activity of 4.07 GBq (IQR 3.95–5.7). In contrast, in 2021–2022, 13% received 4 GBq and 87% received 6 GBq, with a median activity of 6 GBq ( IQR 6–6) (Fig. 1B). A significant difference in variance was observed in administered activity between the two cohorts (Fig. 2B, *P* < 0.01).

In 2021–2022, patients stratified as low risk were treated with 1 GBq of RAI 62.5% of the time compared to only 32% in 2017–2021 (Fig. 1C). No significant difference was observed in low-risk patients, with a median activity of 2.01 GBq (IQR 1.027–3.98) vs 1 GBq (IQR 1–4) (Fig. 2C, *P* = 0.30).

### Outcome

In the prospective cohort, 23 patients had evidence of persistent disease, with neck disease or distant metastasis (one bone and three suprasternal notch) on the initial post-treatment WBS. Recurrence occurred in 10 out of 89 patients (11%) in the prospective cohort; three had a biochemical incomplete response, and seven structural disease. All patients who had recurrence were stratified as either intermediate or high risk of recurrence by our tool. Only four patients had clinically significant disease that warranted further treatment; two were treated with a further activity of RAI; and two underwent lymph node surgery during the 12-month follow-up period.

In the retrospective cohort, 26 out of 393 (6.6%) of patients required further treatment with either RAI or surgery. There was so significant difference in additional treatments between the two cohorts (6.6% vs 4.5%, *P* = 0.628).

## Discussion

This study demonstrates the utility of an online clinical support tool in providing homogenous treatment for patients with DTC. Despite having the 2015 ATA guidelines, our data show there was significant variability in activity prescription in our 2017–2021 cohort – reflecting the degree of noise in this realm. However, when an online support tool was made available, the inter-rater variability between clinicians’ judgments was substantially reduced. This resulted in consistent treatment being administered to patients stratified with similar recurrence risk.

As described by Kahneman *et al*., noise can occur in all fields of medicine, and the effect of noise can be significant (2). Guidelines offer a platform to reduce noise; however, implementing these guidelines can pose challenges in thyroid cancer management (1). First, there is low volume of high-risk diseases, making it challenging for clinicians to gain experience in managing such cases (9). Secondly, clinicians often encounter logistical hurdles in accessing multidisciplinary care, which is most pronounced in rural and remote communities where there are already existing inequalities in thyroid cancer outcomes (10). Finally, there can be lack of confidence in implementing new recommendations, all of which lead to inconsistent practice. In our retrospective cohort, out of 77 patients stratified as high risk by our calculator, six (8%) were treated with only 1 GBq of RAI. Among these six patients, four had sTg levels greater than 30 µg/L (ranging from 33 to 108 µg/L), which were not available at the time of the initial RAI activity selection. This underscores the importance of incorporating unstimulated Tg levels into treatment decision tools. Additionally, the remaining two patients – a 44-year-old with Hürthle cell carcinoma and a 42-year-old with follicular thyroid carcinoma (FTC) – both had more than four foci of vascular invasion. These patients are also classified as high risk by ATA guidelines and would typically be administered a higher activity of RAI, highlighting a discrepancy in treatment practices.

Furthermore, there was significant variability in RAI activity prescription among patients in our retrospective cohort who were stratified as low risk by our tool. Among these patients, 43 out of 85 would also be classified as low ATA risk. While the ATA guidelines are comprehensive, they do not provide specific dosing recommendations for low-risk patients (1). Additionally, the three randomized controlled trials evaluating the utility of RAI in low-risk patients were not published until 2018 (11), 2019 (12), and 2021 (13), respectively. Our findings highlight the slow incorporation of evolving literature into clinical practice. An online clinical support tool could help address this issue by reducing logistical hurdles, ensuring that up-to-date clinical practice guidelines are readily accessible, and facilitating more consistent RAI activity prescriptions.

Noise, characterized by randomness in decision-making, differs from bias, which involves systematic inaccuracy. Evidence-based practice plays a crucial role in reducing bias in treatment. Determining optimal RAI activity for different risk categories remains an evolving field. ESTIMABL1 (11), Hi-Lo study (12), and ESTIMABL 2 (13) established the utility of low activity of RAI in managing low-risk patients. However, optimal activity for patients with intermediate and high risk has not yet been established. For the purposes of this study, we used specific activity recommendations to achieve consistency and found similar recurrence rates in a 12-month period to that published in the literature; with 8 out of 57 (14%) patients in the intermediate-risk group and 2 out of 10 (20%) patients in the high-risk group (1). Further research will be required to determine the optimal activity for different subgroups within intermediate- and high-risk thyroid cancer, in particular considering responses in the context of molecular profile (e.g. BRAF mutated vs RAS mutated). Furthermore, we anticipate that the increasing trend of not administering RAI to low-risk patients has contributed to an increased proportion of high-risk patients (81.5% were intermediate or high risk) in our cohort. This selection bias highlights a limitation of our study: the inability to capture low-risk patients who are not treated with RAI, a topic that will need to be explored in future work. Additionally, application to other centers will be necessary to ensure the widespread feasibility of the tool.

## Conclusion

While optimal risk classification and activity recommendation continues to be established, our data suggest that providing risk stratification and activity recommendation in an easily accessible online tool can reduce noise and variability in activity prescription for patients with DTC.

## Declaration of interest

The authors declare that there is no conflict of interest that could be perceived as prejudicing the impartiality of the research reported.

## Funding

This work did not receive any specific grant from any funding agency in the public, commercial, or not-for-profit sector.

## Author contribution statement

AW – ethics approval, analyzed data, developed predictive risk model, created online clinical support tool, and wrote article. MG – contributed to predictive risk model development, reviewed and edited manuscript. LT – contributed to predictive risk model development, reviewed and edited manuscript. VT – contributed to predictive risk model development, reviewed and edited manuscript. AG – contributed to predictive risk model development, reviewed and edited manuscript. AG – contributed to predictive risk model development, reviewed and edited manuscript. MS – contributed to predictive risk model development, reviewed and edited manuscript. SS – contributed to predictive risk model development, reviewed and edited manuscript. PR – contributed to predictive risk model development, reviewed and edited manuscript. GS – contributed to predictive risk model development, reviewed and edited manuscript. JH – contributed to predictive risk model development, reviewed and edited manuscript. BR – contributed to predictive risk model development, reviewed and edited manuscript. RC-B – review of data analysis, idea for predictive risk model and online clinical support tool, developed predictive risk model, and reviewed and edited manuscript.

## Acknowledgements

The authors acknowledge Dr Adam Aniss the custodian of the thyroid cancer database at RNSH. The authors acknowledge the technical assistance of Alex Shaw of the Sydney Informatics Hub, a Core Research Facility of the University of Sydney.
